# Supplementary material for: WordCommentsAnalyzer: A windows software tool for qualitative research
Source: F1000Res. 2018 Sep 4;7:536. Originally published 2018 May 3. [Version 2] doi: 10.12688/f1000research.14819.2 (PMC6137411; doi:10.12688/f1000research.14819.2)
Supplement: Supplementary file 3 [file f1000research-7-17614-s0002.tgz › b2a79496-4ada-47c4-9682-a99ce867836d.zip!/Eight Iranian Health Journals/Salmand_Iranian Journal of Ageing _14.docx]

© Salmand_Iranian Journal of Ageing . This work is licensed under CC BY-NC 4.0 (https://creativecommons.org/licenses/by-nc/4.0/).

Volume & Issue: دوره 9 - شماره 4

Title: چه مقدار تغییر در آزمون «پنج بار برخاستن و نشستن از روی صندلی» را بایستی تغییری واقعی قلمداد کرد؟

http://salmandj.uswr.ac.ir/article-1-771-fa.html

اهداف: تکرارپذیری نسبی و مطلق از جنبه‌های مهم هر آزمون هستند که مبنای بسیاری از تصمیم‌گیری‌های بالینی قرار می‌گیرند. در بسیاری موارد تنها تکرارپذیری نسبی مبنای تصمیم‌گیری و توجه قرار می‌گیرد درحالی‌که تکرارپذیری مطلق هم از اهمیت بسیاری برخوردار است. مواد و روش‌ها: 11 سالمند ساکن اجتماع در دامنه سنی 65 سال و بالاتر (3/58±69/64 سال) و 20 جوان سالم ساکن اجتماع در دامنه سنی 20 تا 35 سال (4/15±28/80 سال) با استفاده از آزمون «پنج بار برخاستن و نشستن از روی صندلی» دوبار با فاصله زمانی 2 تا 5 روز مورد ارزیابی قرار گرفتند. یافته‌ها: در این تحقیق، شاخص تکرارپذیری مطلق «حداقل تغییر قابل تشخیص سالمندان» در سالمندان 5/29 ثانیه و در جوانان 3/02 ثانیه و ضریب واریانس به ترتیب %27 و %19 به‌دست آمد. نتیجه‌گیری: نتیجه این تحقیق نشان داد که در ارزیابی میزان پیشرفت سالمندان در طی جلسات درمانی یا قضاوت در مورد سیر پیشرفت یا پسرفت توانایی آن‌ها، سالمندان نیازمند تغییر بیشتری نسبت به جوانان هستند تا تغییرات مشاهده شده به عنوان یک تغییر واقعی -و نه تصادفی- قلمداد شود. همچنین سهم خطای تصادفی آزمون "پنج بار برخاستن و نشستن از روی صندلی" در سالمندان اندکی بیش از جوانان بود.

Title: اثربخشی باغبانی درمانی بر سلامت عمومی و عزت‌نفس سالمندان مقیم آسایشگاه

http://salmandj.uswr.ac.ir/article-1-736-fa.html

اهداف: پژوهش حاضر به منظور بررسی اثربخشی باغبانی‌درمانی بر افزایش عزت‌‌نفس و سلامت عمومی سالمندان مقیم آسایشگاه انجام شد. مواد و روش‌ها: روش تحقیق حاضر نیمه‌آزمایشی و طرح آن پیش‌آزمون-پس‌آزمون با گروه کنترل بود. جامعه آماری کلیه سالمندان مقیم آسایشگاه‌های تهران در سال 1392 بودند که 24 نفر از آنان به روش در دسترس به عنوان نمونه تحقیق انتخاب شدند و سپس در دو گروه کنترل و آزمایش به روش کاملا تصادفی گمارده شدند. برای جمع‌آوری داده‌ها از پرسش‌نامه عزت نفس کوپر اسمیت و سلامت عمومی (GHQ) استفاده شد. ملاک‌های ورود کسب نمره پایین در آزمون عزت نفس و سلامت عمومی و ملاک خروج نیز انصراف هر کدام از اعضاء از ادامه همکاری در تحقیق بود. گروه آزمایش در معرض 13 جلسه آموزش باغبانی قرار گرفت و تجهیزات باغبانی توسط محقق در اختیار آن‌ها قرار گرفت. سپس هر دو گروه دوبار پرسش‌نامه‌های مذکور را پس از پایان دوره باغبانی‌درمانی (پس‌آزمون) و سه ماه پس از آن (پیگیری) تکمیل نمودند. تجزیه و تحلیل داده‌ها با استفاده از تحلیل واریانس با اندازه‌های تکراری صورت گرفت. یافته‌ها: نتایج نشان داد که بین میانگین‌های نمرات سلامت عمومی و عزت‌نفس گروه آزمایش در مراحل پس‌آزمون و پیگیری نسبت به مرحله پیش‌آزمون تفاوت معنادار وجود داشت. به عبارت دیگر باغبانی‌درمانی توانسته است سلامت عمومی و عزت نفس سالمندان مقیم آسایشگاه را به‌طور معناداری (0/10>P) افزایش دهد. نتیجه‌گیری: نتایج تحقیق نشان می‌دهد که باغبانی‌درمانی هم باعث افزایش سلامت عمومی و عزت‌نفس سالمندان مقیم آسایشگاه می‌شود و هم به عنوان یک درمان غیردارویی و کم‌هزینه برای سایر گروه‌های بیماران نیز پیشنهاد می‌گردد.

Title: بررسی کیفیت زندگی و متغیرهای دموگرافیک مؤثر بر آن در سالمندان تحت پوشش مرکز بهداشت جنوب تهران در سال 1389

http://salmandj.uswr.ac.ir/article-1-641-fa.html

اهداف: افزایش امید به زندگی و کاهش میزان باروری باعث افزایش سالمندان در سراسر جهان گردیده است. با توجه به این افزایش و مطرح بودن نیازهای خاص این مرحله از زندگی بررسی کیفیت زندگی سالمندان اهمیت می‌یابد. مطالعه حاضر با هدف تعیین کیفیت زندگی سالمندان مرکز بهداشت جنوب تهران و متغیرهای دموگرافیک موثر بر آن صورت گرفت. مواد و روش‌ها: این مطالعه از نوع توصیفی-تحلیلی می باشد. تعداد افراد مورد مطالعه 132 نفر است که به صورت نمونه‌گیری خوشه‌ای انتخاب شدند. گردآوری داده ها به روش مصاحبه ساختاری و ابزار آن پرسش‌نامه خصوصیات دموگرافیک و ابزار بررسی کیفیت زندگی از نوع فرم کوتاه بود. داده‌ها با کمک روش‌های آماری توصیفی و تحلیلی مورد بررسی قرار گرفتند. یافته‌ها: میانگین مجموع نمرات بعد فیزیکی 24/42±54/42 و میانگین مجموع نمرات بعد روانی 24/04±55/19 بود. میانگین سن افراد 6/86±67/98 بود. بین سن و کیفیت زندگی رابطه معکوس و معنی‌داری وجود داشت نمرات کیفیت زندگی در مردان بالاتر از زنان بود. و افراد متاهل نسبت به افراد مجرد کیفیت زندگی بالاتری داشتند. بین سطح تحصیلات و کیفیت زندگی رابطه مستقیم و معنی‌داری وجود داشت. افرادی که با همراه زندگی می کردند نسبت به افراد تنها کیفیت زندگی بالاتری داشتند. رابطه معنی‌داری بین فعالیت بدنی و کیفیت زندگی نشان داده شد. اما بین شاخص توده بدنی و کیفیت زندگی رابطه معنی‌داری نبود. نتیجه‌گیری: نتایج حاکی از متوسط بودن کیفیت زندگی سالمندان و تحت‌تاثیرقرار گرفتن ابعاد کیفیت زندگی توسط عوامل زمینه‌ای و ضرورت برنامه‌ریزی برای ارتقای کیفیت زندگی سالمندان و آگاهی دادن به سالمندان در خصوص فواید اصلاحات رفتاری بر کیفیت زندگی آن‌ها می‌باشد.

Title: تأثیر شش هفته تمرینات تعادلی بر برخی ویژگی‌های کینماتیکی راه‌رفتن در زنان سالمند

http://salmandj.uswr.ac.ir/article-1-685-fa.html

اهداف:‌ هدف از این تحقیق بررسی تاثیر شش هفته تمرینات تعادلی بر برخی ویژگی های کینماتیک راه رفتن در زنان سالمند بود. مواد و روش‌ها: بدین منظور 20 زن سالمند(میانگین±انحراف معیار؛ سن: 4/84±67/72؛ وزن: 6/56±67/27 کیلوگرم؛ قد: 0/036±155 سانتی متر) که تعادل ایستا (به‌وسیله آزمون مقیاس برگ) و تعادل پویا (آزمون برخاستن و رفتن) آن‌ها در حد استاندارد تحقیق بودند، به صورت داوطلبانه در این تحقیق شرکت نمودند. آزمودنی‌ها به‌صورت تصادفی به دو گروه ده نفری کنترل و تمرین تقسیم شدند. سپس از آزمودنی های هر دو گروه یک آزمون مشابه پیاده روی، در طی دو جلسه پیش و پس آزمون به فاصله شش هفته از هم گرفته شد. گروه تمرین در طول این مدت به طور منظم هفته ای 3 جلسه، و در مجموع 18 جلسه تمرینات تعادلی به منظور درگیر کردن سیستم های حسی پیکری، بینایی و دهلیزی از جمله (پریدن از گودال، را ه رفتن هشت انگلیسی، عبور از مانع، راه رفتن با پاشنه پا و ...) را انجام دادند در صورتیکه گروه کنترل فعالیت طبیعی و روزمره خود را دنبال می کردند. در هر دو جلسه پیش و پس آزمون، بعد از نصب مارکرها بر نقاط مورد نظر بدن آزمودنی های هر دو گروه، از آن‌ها خواسته شد تا مسیر7 متری را با سرعت گام‌برداری ترجیحی راه بروند. با استفاده از دستگاه Motion analysis ویژگی‌های کینماتیک راه رفتن شامل طول گام، عرض گام، زمان اتکای دوگانه و سرعت راه رفتن جمع‌آوری و از طریق نرم‌افزار Cortex تجزیه و تحلیل شد. داده‌ها با استفاده از تحلیل واریانس مرکب (2گروه×2زمان) تحلیل شدند. یافته‌ها: نتایج تحقیق نشان داد شش هفته تمرینات تعادلی در زنان سالمند 75-60 سال باعث افزایش معنی‌دار در پارامترهای کینماتیکی راه رفتن ازجمله طول گام 11/33درصد (0/01=P)، سرعت راه رفتن 18درصد (0/04=P) در گروه تمرین، در مقایسه با گروه کنترل شد. در متغیرهای، زمان اتکای دوگانه (0/06=P) راه رفتن و عرض گام (0/48=P)، با وجود کاهش نسبی در گروه تمرین، این تغیرات درگروه کنترل معنی‌دار نبودند. نتیجه‌گیری: نتایج این مطالعه تاثیر شش‌هفته تمرینات تعادلی را در افزایش اندازه طول گام و سرعت راه رفتن در زنان سالمند را تائید نمود. بنابراین برنامه تمرینی مورد نظر بهبود کلی در الگوی راه رفتن را در سالمندان زن، ایجاد کرده است.

Title: بررسی پاسخ برخی از مهم‌ترین پروتئین‌های درگیر در سندروم کا‌چکسیا به فعالیت حاد مقاومتی در افراد سالمند سالم

http://salmandj.uswr.ac.ir/article-1-587-fa.html

اهدف: هدف از ا‌نجام این تحقیق بررسی پاسخ مایوا‌ستاتین پلاسما و هورمون رشدی شبه انسولین (IGF-1) سرم، به عنوان دو تا از مهمترین پروتئین های درگیر در سندروم کا‌چکسیا، به فعا‌لیت حاد مقاومتی در افراد سالمند سالم بود. مواد و روش‌ها: در این مطالعه 11 مرد (سن 1/3±67 سال و kg/m2 25±4/1=BMI) سالمند سالم، به صورت داوطلبا‌نه شرکت داشتند. 72 ساعت پس از تعیین حداکثر قدرت بیشینه (با استفاده از آزمون 1-RM)، آزمودنی‌ها دریک جلسه مقاومتی با شدت 75درصد 1-RM شرکت نمودند. در این پژوهش 2 نمونه خونی در قبل و بلافاصله بعد ازفعالیت مقاومتی از ورید بازویی آزمودنی‌ها گرفته شد. به‌منظور تعیین میزان مایواستاتین پلاسما و IGF-1 سرم از روش الایزا استفاده شد. به منظور تجزیه وتحلیل آماری داده‌ها از آزمون تی تست وابسته استفاده شد. سطح معناداری P≤0/05 در نظر گرفته شد. یافته‌ها: نتایج تحقیق حاضر نشان داد که میزان مایواستاتین پلاسما در پاسخ به فعالیت مقاومتی کاهش معناداری یافت (P=0/0001). همچنین میزان IGF-1 سرم نیز در پاسخ به فعالیت مقاومتی افزایش معناداری یافت (P=0/0001). درهمین راستا نتایج نشان داد که نسبت IGF-1 به مایواستاتین تحت تاثیر فعالیت مقاومتی افزایش معناداری یافت (P=0/001). نتیجه‌گیری: یافته‌های این تحقیق نشان داد که فعالیت مقاومتی ازطریق افزایش میزان IGF-1 وکاهش میزان مایواستاتین موجب افزایش نسبت میزان IGF-1 به مایواستاتین می‌گردد. بنابر یافته‌های این تحقیق به نظر می‌رسد که با تجویز فعالیت مقاومتی می‌توان موجب به‌وجود آوردن تغییراتی مثبت در پروتئین‌های درگیر در سندروم کاچکسیا در افراد سالمند شد. ازاین‌رو احتمال دارد که ازاین طریق بتوان علاوه بر جلوگیری از کاچکسیا از بسیاری از اختلالات فیزیولوژیکی و ناتوانی‌های جسمانی ناشی از کاچکسیا در این افراد جلوگیری نمود. هر چند درک دقیق مکانیسم‌های مربوطه نیازمند تحقیقات بیشتری می‌باشد.

Title: مقایسه سطوح مختلف نگرش مذهبی بر احساس معنا، تنهایی و شادکامی در زندگی سالمندان تحت پوشش سازمان بهزیستی

http://salmandj.uswr.ac.ir/article-1-682-fa.html

اهداف: هدف این پژوهش مقایسه سطوح متفاوت نگرش مذهبی بر احساس معنا، تنهایی و شادکامی در زندگی سالمندان تحت پوشش اداره بهزیستی شهرستان ارومیه بود. مواد و روش‌ها: پژوهش حاضر از نوع توصیفی-تحلیلی بود و نمونه ای به حجم 221 نفر (81 زن و 140 مرد) از سالمندان بالای 60 سال با استفاده از روش نمونه‌گیری طبقه ای انتخاب شدند. پرسش‌نامه نگرش مذهبی خدایاری فرد و همکاران، پرسش‌نامه احساس تنهایی راسل، پرسش‌نامه معنا بخش بودن صالحی و پرسش‌نامه شادکامی آکسفورد در این پژوهش به‌عنوان ابزار استفاده گردید. یافته‌ها: نتایجی که از روش تحلیل واریانس چند متغیری و روش پیگیری شفه بدست آمد، نشان داد که هر چه سالمندان در سطح قوی‌تر نگرش مذهبی قرار داشته باشند، شادکامی و احساس معنا در زندگی آنها نیز بیشتر است(0/05 نتیجه گیری: نگرش مذهبی یک عامل پیشگیری‌کننده جهت کاهش مشکلات سالمندان به شمار می‌رود و بر این اساس مسئولین ذیربط می‌توانند با برنامه‌ریزی‌های مناسب به تقویت باورهای مذهبی سالمندان بپردازند و زمینه‌های لازم جهت شرکت در فعالیت‌های مذهبی را برای سالمندان فراهم کنند.

Title: بررسی تأثیر تمرین ویبریشن کل بدن و تمرین ذهنی بر تعادل مردان سالمند

http://salmandj.uswr.ac.ir/article-1-770-fa.html

اهداف: هدف از انجام این پژوهش تعیین تاثیر تمرین ویبریشن کل بدن و تمرین ذهنی بر تعادل ایستا و پویای مردان سالمند بود. مواد و روش‌ها: جامعه آماری مردان سالمند 60 تا 80 ساله شهر مشهد بودند، که بدین منظور 42 نفر مرد سالمند و سالم، انتخاب و به صورت تصادفی به چهار گروه تمرین ویبریشن (n=12)، ذهنی (n=10)، ترکیبی (n=10) و کنترل (n=10) تقسیم شدند. گروه تمرین ویبریشن تمرینات را به مدت 8 هفته و بر اساس اصل اضافه‌بار با فرکانس 35-30 هرتز و دامنه 5-8 میلی متر انجام دادند. گروه ذهنی نیز به تمرین ذهنی آزمون زمان برخاستن و راه رفتن ‏پرداختند. گروه تمرین ترکیبی مشابه با دو گروه تمرینی ویبریشن و ذهنی به انجام تمرینات پرداختند. تعادل ایستا و پویا به ترتیب با آزمون‌های ثبات وضعیتی و زمان برخاستن و راه رفتن ارزیابی شدند. برای تجزیه و تحلیل داده‌ها از آزمون آماری تحلیل واریانس یک راهه و همچنین آزمون تعقیبی Gabriel استفاده شد (0/05≥α). یافته‌ها: نتایج نشان داد که تفاوت معنی‌داری بین گروه‌ها در تعادل ایستا وجود دارد (0/004=P)، این اختلاف معنی‌دار بین گروه کنترل با گروه‌های تمرین ذهنی (0/005=P) و ترکیبی (0/026=P) است، اما تفاوت معنی‌داری بین گروه کنترل با گروه ویبریشن (0/422=P) و گروه‌های تجربی با یکدیگر مشاهده نشد. در بررسی تعادل پویا نیز مشخص شد که اختلاف معنی‌داری بین گروه‌ها وجود دارد (0/001=P)، این اختلاف مربوط به گروه کنترل با گروه‌های ویبریشن (0/001=P)، ذهنی (0/004=P) و ترکیبی (0/001=P) می‌باشد. علاوه بر این، اختلاف معنی‌داری بین سه گروه وجود نداشت. نتیجه‌گیری: بر اساس نتایج پژوهش حاضر به نظر می‌رسد یک دوره هشت هفته‌ای تمرین ویبریشن، ذهنی و ترکیبی می‌تواند سبب بهبود تعادل پویا، همچنین تمرین ذهنی و ترکیبی باعث بهبود تعادل ایستا در مردان سالمند می‌شود.

Title: ارتباط بین سطح فعالیت بدنی و عوامل خطرزای قلبی‌عروقی در مردان سالمند

http://salmandj.uswr.ac.ir/article-1-742-fa.html

اهداف: پژوهش حاضر با هدف بررسی ارتباط بین سطح فعالیت بدنی و عوامل خطرزای قلبی–عروقی در مردان سالمندان بود. مواد و روش‌ها: جامعه آماری این تحقیق کلیه مردان سالمند شهرستان کرمانشاه بودند. سپس 123 نفر آزمودنی مرد سالمند با میانگین سنی 3/58±63/5 سال، قد 7/83±174/11 سانتی متر، وزن 8/13±84/23 کیلوگرم و شاخص توده بدن 4/2±27/74 کیلو‌گرم بر متر مربع‌ به صورت نمونه‌گیری خوشه‌ای و تصادفی انتخاب شدند. آزمودنی‌ها پرسش‌نامه مربوط به سلامتی، فرم رضایت‌نامه و پرسش‌نامه سطح فعالیت بدنی (Beack) را تکمیل کردند. اندازه‌گیری‌های تحقیق بدین قرار بودند: تعیین وزن بدن، قد، شاخص توده بدن (BMI)، تخمین درصد چربی بدن، نسبت دور کمر به لگن (WHR) و تعیین برخی از عامل‌های خطرزای CHD (کلسترول (TC)، تری گلیسیرید (TG)، لیپوپروتئین کم چگال (LDL)، لیپوپروتئین پر چگال (HDL)، فشار خون سیستولیک (SBP) و فشار خون دیاستولیک (DBP). برای تجزیه و تحلیل داده‌ها از روش‌های آمار توصیفی و استنباطی مناسب شامل آزمون کولموگروف اسمیرنوف و همبستگی پیرسون در سطح معنی داری 05/0 استفاده می‌شود. یافته‌ها: نتایج مطالعه حاضر نشان داد که بین سطح فعالیت بدنی با درصد چربی بدن، شاخص توده بدن (BMI)، نسبت دور کمر به لگن (WHR)، کلسترول (TC)، تری گلیسیرید (TG)، لیپوپروتئین کم چگال (LDL)، فشار خون سیستولیک (SBP) و فشار خون دیاستولیک (DBP) ارتباط معکوس و معنی‌داری وجود دارد. همچنین نتایج نشان داد که سطح فعالیت بدنی رابطه مثبت و معنی‌داری با سطح لیپوپروتئین پر چگال (HDL) داشته است. نتیجه‌گیری: این مطالعه نشان داد که شییوع عوامل خطرزایی قلبی-عروقی و فشار خون بالا در سالمندان بیش از حد انتظار است. همچنین این مطالعه مشخص کرد که افزایش در سطوح فعالیت بدنی می‌تواند در کاهش خطر ابتلا به بیماری‌های قلبی–عروقی موثر باشد، پیشنهاد می‌شود که از طریق راه کارهای مختلف مانند برگزاری کارگاه‌های آموزشی، برگزاری جلسات سخنرانی در مورد فواید فعالیت ورزشی منظم و افزایش مشارکت در فعالیت ورزشی می‌تواند روشی برای ارتقاء سطح سلامتی و کاهش بیماری‌های قلبی- عروقی در آنان باشد.
